# 

**Published:** 1847-02

**Authors:** 


### WESTERN JOURNAL

OF

### MEDICINE AND SURGERY.

### EDITORS.

DANIEL DRAKE, M.D.,

PROFESSOR OF PATHOLOGY AND THE PRACTICE OF MEDICINE IN THE MEDICAL INSTITUTE OF LOUISVILLE:

LUNSFORD P. YANDELL, M.D.,

PROFESSOR OF CHEMISTRY AND PHARMACY IN THE SAME:

THOMAS W. COLESCOTT, M.D.

☞ Our ample experience of the difficulty and uncertainty of collecting from distant subscribers, compels us to decline all orders for this Journal not accompanied by the cash or a satisfactory reference. We were reluctant to adopt this course, but success in our undertaking requires it, and it can result in no material inconvenience to subscribers. All remittances by mail, post paid, are at our risk.

Feb 1847 PRENTICE & WEISSINGER.

The WESTERN JOURNAL OF MEDICINE AND SURGERY is published monthly by the undersigned, at the corner of Main and Fifth streets, Louisville, at $5 per annum, payable in advance. Each number contains 96 pages, making two volumes in the year of more than 550 pages.

Contributions to its pages by the Physicians of the Valley of the Mississippi, addressed to the Editors, are respectfully solicited.

Letters on business, to be addressed, postage paid, to the publishers.

*January*, 1844. PRENTICE & WEISSINGER.

